# Polymeric and Composite Carriers of Protein and Non-Protein Biomolecules for Application in Bone Tissue Engineering

**DOI:** 10.3390/ma16062235

**Published:** 2023-03-10

**Authors:** Dagmara Słota, Karina Piętak, Josef Jampilek, Agnieszka Sobczak-Kupiec

**Affiliations:** 1 Department of Materials Science, Faculty of Materials Engineering and Physics, Cracow University of Technology, 37 Jana Pawła II Av., 31-864 Krakow, Poland; 2 Department of Analytical Chemistry, Faculty of Natural Sciences, Comenius University, Ilkovicova 6, 842 15 Bratislava, Slovakia; 3 Department of Chemical Biology, Faculty of Science, Palacky University Olomouc, Slechtitelu 27, 783 71 Olomouc, Czech Republic

**Keywords:** biomolecules, hormones, flavonoids, lipids, growth factors, protein amino acids, osteopontin, bone sialoprotein, osteocalcin, osteonectin

## Abstract

Conventional intake of drugs and active substances is most often based on oral intake of an appropriate dose to achieve the desired effect in the affected area or source of pain. In this case, controlling their distribution in the body is difficult, as the substance also reaches other tissues. This phenomenon results in the occurrence of side effects and the need to increase the concentration of the therapeutic substance to ensure it has the desired effect. The scientific field of tissue engineering proposes a solution to this problem, which creates the possibility of designing intelligent systems for delivering active substances precisely to the site of disease conversion. The following review discusses significant current research strategies as well as examples of polymeric and composite carriers for protein and non-protein biomolecules designed for bone tissue regeneration.

## 1. Introduction

**Figure 1:**
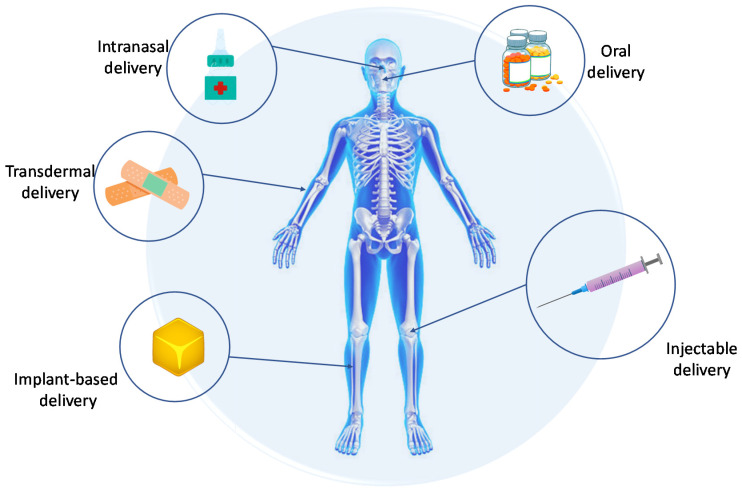
Strategies for delivery of biomolecules.

Drug carriers are biologically safe tools for transporting molecules for nutraceutical, pharmaceutical, and cosmetic applications, i.e., in fields of high industrial and scientific interest. According to the report Drug Delivery Systems Market Size, Share and COVID-19 Impact Analysis (Report ID: FBI103070), the global drug delivery systems market size was valued at USD 39.33 billion in 2022 and is projected to grow to USD 71.75 billion by 2029, exhibiting a compound annual growth rate of 9.0%. Only in North America, this market was valued at USD 14.15 billion in 2021. These costs are associated with the need for high financial outlays for research and investment. An estimated 10,000 molecules are generally rejected before one is selected as marketable [1,2]. In recent years, increasing emphasis has been given to using polymeric or composite materials as carriers for active substances, attempting to develop a biomaterial that will carry a therapeutic effect at a specific location in the organism. The reason for this is that the total therapeutic benefit of a drug or other active substance is not proportional to its potency in vitro. Under physiological conditions, active substances meet several barriers, such as aggregation, insolubility, degradation, or impermeability of vascular endothelial cell layers, which are responsible for the drug’s short half-life in vivo as well as non-specific distribution in tissues or poor tissue penetration. Consequently, there is a risk of adverse effects, such as immunogenicity and off-target toxicity [3,4]. The main goal of designing biomaterials for active substance delivery is to release pharmacologically active biomolecules for specific drug action at an optimal rate and dose. Thus, it is possible to personalize such a system, adapting it to the needs of a particular patient (taking into account his/her gender, height, and age) [5]. There are many methods of delivering drugs and active biomolecules, not only orally, but also transdermally or inhaled (Figure 1). However, in the case of bone regeneration, the subject of this review, innovative composites and matrices (i.e., implants) or injectable delivery systems have the most significant potential [6].

However, it is important to consider that drug carriers can be classified by their specific properties, including their shape, dimensions, application, and especially the way the drug content is delivered [1]. The oral administration system is the conventional and most commonly used method of drug supply. It is based on the oral dissolution and/or diffusion process. However, this method can adversely affect cellular pH and microflora in the stomach and involves the systemic distribution of the substance throughout the body and low absorption [7,8]. Other types of administration are passive or active delivery. In the first case, the release of the drug and thus the delivery of the therapeutic dose at the targeted site is a result of the natural response of the cells. In active delivery, an external stimulus must be added to the system to release a dose of the active substance exactly when it is required. These instances regard the case in which the carrier has already been administrated to the target tissue [9]. Delivery by injection involves delivering the product subcutaneously using a needle. However, a major limitation is possible infection and trauma caused by needles used in syringes [10,11]. Biomolecules and drugs are also delivered transdermally. It is a painless method of delivery by applying a drug formulation onto healthy and intact skin. From such a carrier, a substance can penetrate the body passively, through gradient diffusion, or actively, through artificially induced penetration by means of an electric field, heat, or ultrasound [12,13]. Delivery can also be provided via the intranasal route. Such administration of active substances is characterized by rapid delivery considering the large surface area and rapid systemic adsorption [14].

There are several types of drug carriers. These include nanoparticles, nanotubes, liposomes, dendrimers, and hydrogels. They differ in shape and production method [1]. Nanoparticles have been extensively used as carriers for the delivery of chemicals and biomolecular drugs, such as anticancer drugs and therapeutic proteins [15]. They are divided into two groups, nanocapsules and nanospheres, and have many different applications [16]. Due to their small size, nanoparticles can quickly dissolve in the bloodstream and reach a specific site, transporting insoluble drugs. Nanoparticles based on biopolymers, such as proteins, are often used. Not only do they exhibit low toxicity and biodegradability, but they also are easily subjected to surface modification and are not immunogenic [17,18]. However, it is important to mention that metallic nanoparticles are the subject of intense research due to their potential health risks, given the danger of metal accumulation in the organism [19]. Another type of carrier with a significantly small size is nanotubes. They assume a cylindrical shape and are hollow inside. Nanotubes are filled with drugs or other active substances usually by functionalizing the carbon skeleton. Most often, they are used in anticancer therapy. However, information about their cytotoxicity still needs in-depth research [20,21]. On the other hand, hydrogels are a safe alternative to drug delivery systems with high application potential. Depending on the substrates selected for their synthesis, it is relatively easy to control their physicochemical properties or degradation rates. In general, these systems are constructed from distilled water and a polymeric gelling agent. They exhibit a high capacity to absorb liquids into their polymer network. Hydrogels have the ability to protect loaded drugs from the external environment, such as the acidic pH during oral administration [22,23,24]. Other typical polymeric carriers are dendrimers, characterized by a branched, three-dimensional structure. Their shapes can be hexagonal or cubic. The peculiar structure of dendrimers is the reason for the presence of free spaces, so-called cavities, in their molecules; such cavities can be used as specific pockets, in which various molecules can be placed by encapsulation. After fulfilling its role and releasing the active substance, it is usually excreted in the urine and/or biodegraded [25,26,27]. However, the most widely known and used carriers are liposomes. They are small spherical structures made of one or more phospholipid double layers. They exhibit great biocompatibility, but the main advantages of using liposomes are their increased stability and reduced toxicity of the encapsulated drug. Since the layers of liposomes are very similar to cell membranes, they are also characterized by the possibility of direct fusion [28].

Polymers are proving to be increasingly important in the carriers of various drugs. One such active substance is doxorubicin (DOX), which has been encapsulated in polymer micelles based on PEG-*b*-poly(DPA-co-DTM) or PEG-*b*-poly(DBA-co-DTM) copolymer. The micelle size increased after antibiotic loading by about 30% for the first copolymer and by about 21% for the second copolymer. The release kinetics indicate accelerated drug release at lower pH values, which may have potential applications in biomedical therapy [29]. The same active ingredient was also encapsulated into amphiphilic star copolymers with a HyperMacs core, which was prepared from disulfide-bond-containing AB2 polycaprolactone (PCL) macromonomers and then grafted with polyethylene glycol (PEG). As a result of the self-organization of the copolymers, the resulting spherical micelles in an aqueous medium were characterized by their ability to be reduction-cleavable in a reductive environment. In addition, in vitro studies showed a slow release of DOX in PBS, but a rapid release in a reducing medium, providing the possibility of using the resulting micelles for the triggered release of the anticancer drug [30]. Furthermore, doxorubicin was also used to form a magnetically reactive multifunctional magnetic vortex of Fe_3_O_4_@PVP@DOX with a unique domain structure. Interestingly, in vivo animal studies demonstrate that the resulting magnetic vortex nanoplatform can successfully perform magnetic demand response of heat therapy and magnetic-field-induced release of the DOX drug. This results in a synergistic effect in inhibiting tumor growth without any side effects, which is a highly desirable feature when developing this type of material [31]

The presented review demonstrates various methods of delivering selected protein biomolecules (such as growth factors, amino acids, osteopontin, osteocalcin, and osteonectin) and non-protein biomolecules (such as hormones, flavonoids, and lipids), using polymeric and composite biomaterials designed for bone tissue regeneration. The composition of the materials and their test results are discussed. Table 1 summarizes the protein biomolecules discussed in this review, and Table 2 summarizes the non-protein biomolecules.

## 2. Protein Biomolecules

### 2.1. Growth Factors

Naturally occurring substances capable of stimulating cell proliferation, wound healing, and sometimes also cell differentiation are growth factors (GFs). Usually, they are secreted proteins or cytokines that are important for regulating various cellular processes [32]. They act as signaling molecules by creating a complex signaling network between cells [33]. GFs mediate a variety of regulatory functions. They affect wound healing, cell proliferation, and migration as well as local regulation of the immune system. In addition, they can also bind to receptors in the cell membrane and act as stimulators of cell growth and metabolic functions [34]. A single GF may have to regulate different cell types, inducing a range of cellular functions in different tissues. There are three classes of tasks for GFs:

- Autocrine tasks: a specific GF affects a cell of its own origin or cells of identical phenotype. In this case, a GF produced by an osteoblast can influence the activity of another osteoblast;
- Paracrine tasks: a specific GF influences neighboring cells;
- Endocrine tasks: a specific GF floats on phenotypically different, distant cells. In this case, the GF secreted in the nervous tissue of the central nervous system can induce osteoblast activity [35,36].

GFs have important applications in wound healing, skin tissue engineering, cartilage tissue engineering, and bone tissue engineering. There are numerous classes of these biomolecules. They are characterized by different specific biological features, so their clinical applications also vary [32]. This part of the review will focus on different delivery methods of specific growth factors used in the bone healing process.

#### 2.1.1. TGF-β

The transforming growth factor-β (TGF-β) superfamily includes a diverse range of proteins, such as TGF-β1–3, and one of the major proteins in skeletal system regeneration—bone morphogenetic proteins (BMPs) [37]. Different types of ceramics are used to provide biomimetics. Mesoporous calcium phosphate ceramics prepared by soft and hard templating were loaded with TGF-β1, and kinetic release in vitro was studied. The ceramic phase was modified by the adsorption process. Based on the results, from 0 to 50% of the initial TGF-β1 loading was released in a culture medium after 3 to 6 days [38]. Using the cryogenic 3D printing technique, bi-layered osteochondral scaffolds were designed. Osteogenic peptide/β-TCP/poly(lactic-co-glycolic acid) water-in-oil composite emulsions were printed into a hierarchically porous subchondral layer, and poly(lactic acid-co-trimethylene carbonate) water-in-oil emulsions were printed into thermal-responsive cartilage frame on top of the subchondral layer. To form the cartilage module, scaffolds were dispensed with TGF-β1 loaded type I collagen (Col) hydrogel [39]. TGF was likewise used to compose three-dimensional (3D) scaffolds based on silk fibroin (SF) and chitosan (CS) (TGF-β1-SF-CS) [40]. Proliferation and chondrogenic differentiation of rat bone marrow-derived mesenchymal stem cells (MSCs) were also evaluated in relation to gelatin (Gel) hydrogel microspheres. Gel was dehydrothermally cross-linked in different conditions in a water-in-oil emulsion state to obtain Gel hydrogel microspheres with different water content. Microspheres were then loaded with TGF-β1. The obtained results suggest that the designed biomaterial functions well both as the scaffold of MSC and the matrix of TGF-β1 release [41]. To increase the bioactivity of the biomaterial, TGF-β1 was combined with vascular endothelial growth factor (VEGF). The proteins were loaded into the reservoir fabricated from nanocrystalline calcium sulfate hemihydrate (nCaS), hydroxyapatite (HAp), and calcium sulfate hemihydrate (CaS) (nCaS/HAp/CS/TGF-1/VEGF). In vitro studies on the release of growth factors from the nCaS/HAp/CS cement were conducted for 28 days [42]. Applications combining two or more growth factors are more favorable due to their additive or synergistic effects on bone formation. TGF-β1 and insulin-like growth factor 1 (IGF-1) are particularly interesting, as they are highly expressed during bone growth [43]. Thus, TGF-β1 and IGF-1 were incorporated into a hydrogel Gel scaffold and evaluated in a rat tibia segmental defect model. The release of growth factors resulted from hydrogel biodegradation [44]. Cylindrical plasma-sprayed porous-coated implants made of titanium alloy (Ti_6_Al_4_V) were modified with the addition of TGF-β1 and IGF-1 by incubating them in polylactic acid (PLA) solution with dissolved growth factors. Locally delivered growth factors enhance mechanical fixation and osseointegration; however, the release kinetics was not investigated [45]. The same biomaterial was later enriched with a plasma-sprayed HAp coating. As a result, due to the ceramic phase in the biomaterial, bone growth was 3-fold higher, and the local release of TGF-β1 and IGF-1 stimulated gap healing during in vivo studies [46]. Similar studies involved the use of HAp coating modified with TGF-β1 and IGF-1, which was investigated as a fusion cage to improve the results of cervical intervertebral fusion and to treat the goat cervical spine, an interbody fusion model. The obtained results suggest that such coating can enhance bone fusion [47]. Another protein that TGF was coupled with is stromal-derived factor-1α (SDF-1α). It was loaded into a silk fibroin-porous Gel scaffold. The sustained release of biomolecules occurred through in vitro degradation of the polymer matrix [48]. Although TGF-β1 is the most commonly used growth factor of the TGF family, there are also links to other variants. TGF-β2 and growth/differentiation factor 5 (GDF5) as well as their combinations were loaded into silk-fibroin scaffolds, and human primary adipose-derived MSCs were cultured on them [49]. Biodegradable poly(ether carbonate urethane)-urea (PECUU) was used to culture TGF-β3-mediated bone marrow stem cells, and their gene expression profile was investigated [50]. Another polymer matrix used as a TGF-β3 carrier is an electrospun CS-coated PCL fiber scaffold. However, the release kinetics of TGF–β3 in vivo is not known [51]. 

#### 2.1.2. Combination of TGF-β and BMP-2

An interesting combination is the inclusion of TGF and bone morphogenetic protein-2 (BMP-2) in one biomaterial, as BMP is a growth factor, a protein that induces bone formation and osteoblast differentiation. A conically graded scaffold of CS-Gel hydrogel/poly(lactic-co-glycolide) (PLGA) was facilely prepared and loaded with GFs. The CS-Gel hydrogel phase was modified with TGF–β1 to promote chondrogenesis, whereas the PLGA scaffold was loaded with BMP-2 for osteogenesis. The release rate of GFs and the behavior of the obtained materials in vitro and in vivo were determined. The results show that the conically graded transition from the hydrogel to the PLGA scaffold and graded variation in the amount of growth factors from TGF-β1 to BMP-2 benefited the cartilage–bone interface reconstruction [52]. Another way was to combine PLA with PEG and HA to investigate this composition as a carrier for BMP-2. ELISA investigated the release kinetics of BMP-2 from the composites. Thirty-six male Sprague Dawley rats underwent posterolateral spinal fusion on L4–L5 with three different doses of BMP-2. ELISA demonstrated the sustained release of BMP-2 until day 21 [53]. As PLGA and Gel demonstrate good properties as drug carriers, they have also been combined with collagen to create PLGA-Col-Gel electrospun nanofiber membranes loaded with E7-BMP-2. E7-BMP-2 is a bone morphogenetic protein-2-mimicking peptide providing a better economic substitute for human protein [54]. However, the effect of a membrane made out of clear type I Col soaked with BMP-2 for the treatment of the bone system was also determined [55]. To achieve a more biomimetic and bone-like structure, biomaterials containing the ceramic phase are created. Therefore, calcium-deficient hydroxyapatite (CDHAp) was used to create a CDHAp/COL-based bio-ceramic scaffold as a BMP-2 carrier. It was evaluated in vivo in a rat calvarial critical-sized bone defect model. A sustained release of protein was observed for 35 days [56]. However, combinations with stoichiometric HAp of micro- as well as nanometric diameter are more frequent. HAp nanoparticles were soaked in BMP-2 and then suspended in a Col matrix to form a scaffold [57]. Although more frequently, HAp/Col scaffolds are first created, which are then modified with protein by immersing it in its solution. Protein then binds to both components. Scaffolds may be composed of nHAp [58] as well as ceramics of a larger diameter [59,60,61,62] and also enriched with CS microspheres loaded with BMP-2 [63]. Besides scaffolds, organic-nonorganic biomaterials such as membranes [64], coatings [65], and pastes [66] are also developed. In all cases, the release of biomolecules was possible due to the specific polymer structure of Col as well as the porous nature of HAp. 

#### 2.1.3. Combination of BMP-2 and FGF

As mentioned, applying two or more GFs can provide a better therapeutic effect due to the synergy. An example is the cooperation of BMP-2 with fibroblast growth factor (FGF), as they both regulate the spontaneous repair of bone defects. Therefore, HAp/Col scaffold discs were prepared as previously, enriched not only with BMP-2 but also with FGF-2. The obtained in vivo results confirmed that this combination has the potential to increase bone healing in old mice [67]. An interesting project is a coating composed of a biomimetic calcium phosphate layer that is applied to a synthetic bone graft covered with a poly-l-lysine/poly-l-glutamic acid polyelectrolyte multilayer (PEM) film and GFs [68]. Another type of biomaterial binding these two GFs is poly-l-lactic acid (PLLA) core–PLGA shell double-walled microspheres loaded with BMP-2 and FGF-2. It was demonstrated that they enable the release of multiple growth factors according to specific requirements [69]. 

#### 2.1.4. Combination of BMP-2 and PIGF-2

**Figure 2:**
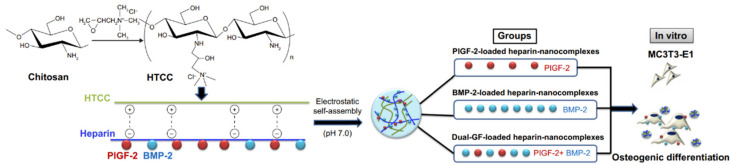
Schematic illustration of the fabrication mechanism and in vitro osteogenic effect of PlGF-2-/BMP-2 nanocomplexes loaded with heparin-HTCC. Reprinted from [71], Copyright 2016 Dove Medical Press Limited.

Also relatively common is the combination of BMP-2 with placental growth factor-2 (PlGF-2), a member of the VEGF subfamily [70]. They were used to create PlGF-2/BMP-2-loaded heparin–*N*-(2-hydroxyl)propyl-3-trimethyl ammonium CS chloride (HTCC) nanocomplexes which have been analyzed to determine the osteogenic effect. Figure 2 illustrates schematically the mechanism of fabrication and in vitro osteogenic effect of the nanocomplexes. Dual delivery of PlGF-2 and BMP-2 could be used to improve osteoregeneration [71].

#### 2.1.5. Combination of BMP-2 and VEGF

However, most commonly, to increase therapeutic effect, BMP is combined with VEGF. VEGFs are responsible for the regulation of vascular development, angiogenesis, and lymphangiogenesis by binding to several receptors [72]. The use of polymer carriers alone and their composites with ceramics has been reported in the literature. An example of the first group is the material consisting of PLGA microspheres loaded with BMP-2 embedded in a poly(propylene) scaffold surrounded by a Gel hydrogel loaded with VEGF for sequential release [73]. Another project incorporated Gel using PLGA/Gel electrospun nanofibers scaffolds combined with BMP-2/VEGF. The quantity of the final release of GFs in an in vitro environment was relatively high, around 80%. However, the release of GFs was not the same and was slower for BMP-2 [74]. Similar results were obtained for GFs delivered by PLA-PEG-PLA block copolymers. The BMP-2/VEGF modification was carried out by soaking, and the release of the substance occurred as a result of polymer degradation [75]. Combinations with bioactive HAp include microspheres of *O*-carboxymethyl CS used as a drug carrier to construct a compound sustained-release system loaded into HAp/Col scaffold [76]. The nanoform of ceramic is presented as a Col-nanoHAp (nHAp) scaffold, where GFs are transported in the Col matrix [77]. Another case includes the production of SF-nHAp biomaterial, where GFs are encapsulated in SF. The modification with BMP-2/VEGF took place via chemical covalent bonding and physical adsorption. However, there is a significant difference between the release kinetics of VEGF and BMP-2 [78]. The mixture of HAp and β-tricalcium phosphate is a specific kind of ceramic and is called biphasic calcium phosphate (BCP). It was combined with nanocellulose loaded with GFs to create sponge-like scaffolds.

Interestingly, the sustained dual release of GFs was less than in previous cases, although it did not show a satisfactory effect on bone formation [79]. Equally curious is the use of calcium phosphate cement (CPC) scaffolds which consist of a mixture of β-tricalcium phosphate, dicalcium phosphate anhydrous, precipitated HAp, and nHAp. They were enriched with PLGA microspheres loaded with GFs. This project has been evaluated for avascular necrosis of the femoral head, and the research suggests that BMP and VEGF in this combination may slow or even reverse the pathological process of femoral head necrosis [80].

#### 2.1.6. VEGF

Nonetheless, VEGF provides a remarkable therapeutic effect in targeted therapy, both in synergy with BMP and on its own, as evidenced by many published studies. It can be supplied with a carrier from a biodegradable PLGA scaffold enriched with GF. The freeze-drying technique mainly obtains this kind of porous foam. Such a structure ensures a sustainable release of substances [81]. A similar study with biomimetic PLGA microsphere scaffolds loaded with VEGF led to similar results [82]. Another more sophisticated polymeric solution to control the growth factor release is an alginate/CS/PLA system. In this material, VEGF was first encapsulated in alginate microspheres via the emulsification method and then combined with freeze-dried scaffolds. GF releases through the degradation of the polymer under in vivo conditions [83]. As shown previously, the ceramic phase in biomaterials for bone regeneration improves biomimetic properties. Hence, it is often added as one of the substrates. The foam casting method produced a high porous β-TCP coated with a thin layer of PLGA containing an encapsulated growth factor [84]. A more extensive project with a VEGF delivery system consisting of PLGA microspheres involved a 3D Gel/alginate/β-TCP scaffold. During the in vitro study, a 50% increase in alkaline phosphatase activity was observed due to VEGF release, which is a promising result [85]. Another type of ceramics used is bioactive glass. It was used to cover PLGA scaffolds with incorporated VEGF. In this case, substrate coating with this bioactive glass offers an inductive component through material degradation [86].

**Figure 3:**
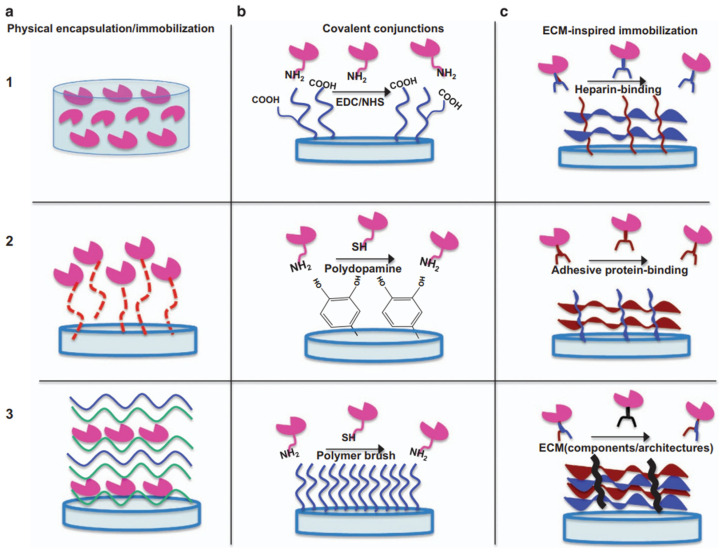
Approaches for the immobilization/encapsulation of growth factors (GFs) to biomaterials. (**a1**–**a3**) Physical immobilization techniques. (**b1**–**b3**) Non-selective covalent immobilization of GFs through their functional residues. (**c1**–**c3**) Extracellular matrix (ECM)-inspired immobilization reactions used for the orientation of GFs on the surfaces of biomaterials. Reprinted from [91], Copyright 2017 Springer Nature Limited.

Nevertheless, among all types of ceramics, the most attractive is HAp. Polymers of natural origin include Col and Gel, representing a denatured form of Col. Using Col and nHAp, a porous scaffold was produced, and the freeze-drying method was used for this purpose. The entire scaffold, including the ceramic phase, was decorated with VEGF [87]. A comparable solution was employed for a ceramic-polymer cryogel composite, where the polymer phase was represented by Gel. The biomaterial was enriched with GF by the microinjection method. However, the release kinetics were not investigated [88]. Silicon-substituted HAp microporous scaffolds were prepared via robocasting. GF was modified through non-covalent binding via incubation in a specific solution. The bone regeneration capability of this biomaterial has been evaluated in vivo using an osteoporotic sheep model, and the obtained results confirmed that they represent suitable bone grafts [89]. An attractive solution using the metallic phase combines Ti_6_Al_4_V-ELI macroporous scaffolds coated with silicon-substituted HAp and enriched with VEGF. For this purpose, electron beam melting and dip coating methods were used. Adsorption and immobilization of vascular endothelial growth factor on scaffold surfaces were carried out through non-covalent binding by incubation. However, a deficient level of GF was observed after the initial time [90]. The development of tissue and material engineering has led to the formulation of three strategies for immobilizing GFs in biomaterials (Figure 3). The simplest method of encapsulating GFs is physical encapsulation (Figure 3(a1)), which involves incorporating GFs into a 3D polymer matrix by mixing factors inside the polymers before the gelation or solidification process. Physical adsorption (Figure 3(a2)) has attracted a lot of attention in recent years; however, this method is associated with poor control of the active substance delivered and inefficient retention of stable soluble proteins. A layer-by-layer (Figure 3(a3)) technique is an alternative to direct adsorption, as it provides better control over the spatial as well as temporal distribution of the active ingredient. This method most often relies on electrostatic interactions between oppositely charged polyelectrolytes and GFs to deposit functional polymer coatings on surfaces of different shapes and compositions. The technique involves chemical/enzymatic reactions between proteins and functionalized surfaces, offering significant control over the amount, orientation, retention, and distribution of GFs on the carrier. When a GF is chemically bound to biomaterials, its desorption rate is controlled by enzymatic or hydrolytic cleavage of the chemical bond. Carbodiimide coupling immobilization (Figure 3(b1)) is most commonly used due to its simplicity of execution, low cost, and mild reaction conditions. Polydopamine layers are also used (Figure 3(b2)), exhibiting strong adhesion properties to nearly all types of surfaces. However, depending on the type of GF and the carrier, other compounds are also used (Figure 3(b3)), such as dextran or streptavidin. The last approach is extracellular matrix (ECM)-inspired immobilization of GFs. In these techniques, taking into consideration natural interactions between ECM and GFs, heparin-based binding, adhesive protein-binding and ECM component and hierarchical structure-based binding are distinguished [91].

#### 2.1.7. Combination of VEGF and PDGF

Other synergistic effects were investigated between VEGF and platelet-derived growth factors (PDGFs). PDGFs are a family of homo- or heterodimeric growth factors, responsible for enhancing the proliferation of fibroblasts and the production of extracellular matrix by these cells [92,93]. However, they are also involved in the process of bone regeneration; thus, a brushite–CS system with incorporated VEGF and PDGF was designed. PDGF was incorporated in the liquid phase during scaffold formation, and VEGF was encapsulated in alginate microspheres and pre-included in small cylindrical CS sponges. The kinetic release was investigated in both in vitro and in vivo conditions in relation to male New Zealand rabbits [94].

#### 2.1.8. PGDF

PDGF-BB on its own was applied in CS sponges. Modification occurred via soaking in GF solution, and the structure was freeze-dried. The release of the active substance occurred because of matrix degradation and, as a result, induced new bone formation [95]. In the more extended model, the CS sponge was enriched with chondroitin-4-sulfate (ChoS) to ensure steady release. The obtained results confirmed that the release of PDGF-BB from the ChoS–CS sponge significantly enhanced osteoblast proliferation [96]. Recombinant human platelet-derived growth factor was combined with Col matrix to ensure guided bone regeneration in in vivo conditions. The results suggest the accuracy of this project; however, the release kinetics have not been presented [97]. The method was also successfully applied to the functionalization of silk fibers (SF). These 3D textile implants were manufactured via an additive manufacturing approach [98]. Last but not least, SFs were combined with bioglass, PDGF, and the above-mentioned BMP-7 to create a mesoporous scaffold. Once again, the synergistic effect resulted in satisfactory results thanks to the sustained release of GFs [99].

#### 2.1.9. FGF

The family of fibroblast growth factors (FGFs) is especially involved in the process of angiogenesis and wound healing but also in embryo development. A defining property of FGFs is that they bind to heparin and heparan sulfate [100]. Apart from the connection to BMP-2 described above, FGF was also utilized as the only protein modifying a HAp/Col scaffold. Abundant bone and cartilage regeneration were observed; however, the release kinetics has not been studied due to the rapid degradation of the biomaterial in in vivo conditions [101]. Other carriers assumed no use of the ceramic phase, focusing only on the properties of polymers. Therefore, the capacity of the hydrogel matrix of poly(2-hydroxyethyl methacrylate) copolymerized with 2-vinyl pyrrolidone to release FGF-2 was evaluated. The release of protein from the copolymer occurred during the swelling process. After this process, FGF-2 was active, which means it was not damaged during polymerization and sterilization [102]. Other polymers, such as PLA, are also used to design scaffolds [103] or manufacture nanosheets [104]. An alternative to solid biomaterials that degrade in a biological environment is injection materials that demonstrate a better cavity fit and allow easy application by injection compared to traditional scaffolds. Therefore, an interesting solution was designed to use a hydrogel composite of chitin/PLGA loaded with FGF-18 and CaSO_4_ for guided bone regeneration as well as enhancing the ability to support osteogenic differentiation [105].

#### 2.1.10. NGF

Furthermore, nerve growth factor (NGF) was experimentally used in targeted skeletal therapy, although it is responsible mainly for the development and survival of certain sympathetic as well as sensory neurons in both the central and peripheral nervous systems [106]. For targeted skeletal therapy, NGF was supplied in Col/nHAp/κ-carrageenan gel in the rabbit model of mandibular distraction osteogenesis [107].

### 2.2. Protein Amino Acids

Although amino acids (AA) are not strictly classified as protein biomolecules, they constitute a broad group of organic compounds, among which protein amino acids are distinguished, i.e., those that are part of proteins and are connected by peptide bonds [108,109]. For this reason, this part of the review will describe the delivery system of the protein amino acids that were experimentally used and described in the regeneration of the skeletal system.

#### 2.2.1. RGD Peptide

**Figure 4:**
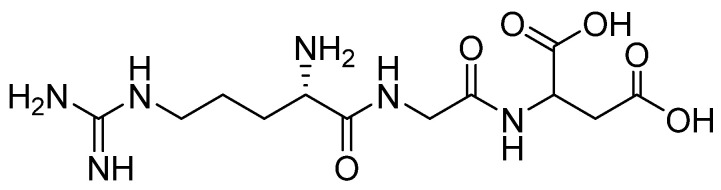
Structure of RGD (Arg–Gly–Asp) peptide.

One of the most commonly used peptides to functionalize biomaterials is an arginine-glycine-aspartic acid sequence called RGD peptide (Arg–Gly–Asp) (Figure 4). It is present in several proteins essential for bone regeneration, such as Col, fibronectin, bone sialoprotein, and osteopontin [110,111]. It is a cell adhesive peptide. Furthermore, various studies have demonstrated that RGD is a promoter of osteogenic differentiation in in vitro conditions and is able to stimulate in vivo bone formation [112]. Therefore, this specific sequence is experimentally combined with other biomaterials to enhance the therapeutic effect. An example is the modification of a ceramic-polymer composite, where the polymer phase was composed of Col and the ceramic phase of bone cement powder, which is a mixture of 58 wt% α-TCP and 24 wt% dicalcium phosphate (DCPA). The biomaterial was enriched with the RGD sequence by impregnation after the subsequent phosphoserine/RGD merger. The kinetics of compound release have not been studied, although under in vivo conditions, RGD appears to lead to increased bone formation around HA/Coll composite cement [113]. Another two-phase project involved creating bioresorbable PLGA/nHAp porous scaffolds decorated with this AA. The scaffold was modified via immersion in RGD solution, and obtained scaffolds were implanted into rabbits to observe preliminary application in the regeneration of mandibular defect [114]. Moreover, biphasic calcium phosphate was used to create BCP/CS scaffolds. These were modified with Arg–Gly–Asp and the aforementioned BMP-2 to evaluate their synergistic effect. RGD was covalently immobilized on the composite scaffold via EDC/NHS reaction. GF was first enclosed in bovine serum albumin nanoparticles and then immobilized on the scaffold surface [115]. For comparison, an example of soft biomaterial providing the RGD sequence is oligoPEG fumarate hydrogel. The presented study investigated the differentiation and mineralization effect of marrow stromal cells (MSCs) cultured in media [116]. Another good candidate material for cartilage tissue regeneration is poly(propylene fumarate) (PPF) due to its excellent mechanical properties during its degradation. Using micro-stereolithography, 3D printing PPF bioinks with immobilized RGD were created. The amino acid sequence’s release depended on the PPF degradation rate [117]. Another soft material is cell–polymer constructs, created out of PEG hydrogel with encapsulated human mesenchymal stem cells (hMSCs) enriched with RGD sequence. In this case, an additional function of the peptide is to increase cell survival [118]. A similar model included encapsulating human embryonic stem cells (hESCs) into RGD-loaded PEGDA hydrogels. As before, the release of the active substance and cells occurred due to the degradation of the polymer matrix [119]. In another study, osteogenic precursor cells (OPC) were placed on PLA films and scaffolds decorated with RGD. However, PLA was firstly surface treated with NH_3_ plasma. The obtained results suggest that this material with RGD significantly enhanced osteogenic cell attachment and differentiation into bone cells [120]. An example of another biomaterial is nanofibers. Arg-Gly-Asp was immobilized onto the electrospun PLGA nanofiber mesh surface to mimic an extracellular matrix structure. Sustained release of AA occurred via the biodegradation of polymer fibers [121]. Polydopamine (PDA) is a polymer that also undergoes degradation in a biological environment. It was used to compose hybrid ZnO/PDA/arginine-glycine-aspartic acid-cysteine (RGDC) nanorods prepared on titanium (Ti) implants. In this case, the RGD sequence was additionally enriched with cysteine amino acid, which also belongs to protein amino acids. These combinations not only enhance osteoinductivity but also have antibacterial properties [122]. For comparison, cysteine was incorporated into the PEG/HAp coating on a titanium alloy. The degradation of the polymer allowed the release of the amino acid from the material [123]. However, *N*-acetyl-l-cysteine, which is an l-cysteine derivative, is used in such biomaterials instead.

#### 2.2.2. Arginine

**Figure 5:**
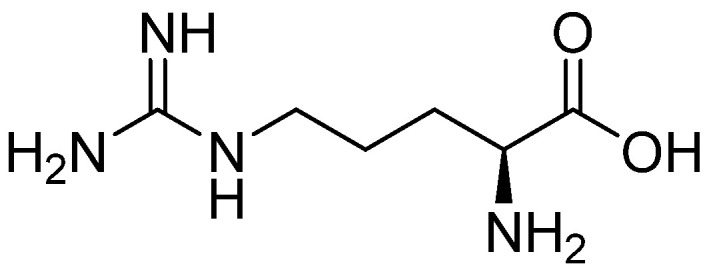
Structure of l-arginine.

Since arginine (Figure 5) belongs to protein amino acids, it is used not only in the form of RGD sequence but also by itself, especially since it encourages cell attachment, proliferation, and differentiation on HAp surfaces. Therefore, composites similar to those already mentioned are created. A bone-like nanostructure was obtained by using nHAp and Col. The nanostructure was then enriched with l-arginine [124].

An example of soft materials without ceramic reinforcement is a controlled-release system slowly releasing arginine–CS/plasmid DNA nanoparticles encoding the aforementioned BMP-2 gene (Arg-CS/pBMP-2 NPs). The Arg-CS/pBMP-2 nanoparticles were then encapsulated into PELA microspheres which work as the controlled-release carrier [125]. An interesting aspect is the application of magnetism and the integration of magnetic nanoarchitectures into synthetic/natural scaffolds. Thus, CS-based scaffolds containing dextran-grafted maghemite nanoarchitectures (DM) and functionalized with l-arginine were manufactured. The obtained results suggest that the simultaneous release of DMs and arginine conferred interesting properties toward osteoblast-like hMSCs, differentially stimulating their proliferation [126]. To promote osteoinductivity, a bioactive hydrogel on the basis of arginine-based unsaturated poly(ester amide) and methacrylated hyaluronic acid (HA) was developed via the photo-crosslinking method. Research has indeed confirmed the stimulation of bone formation processes; however, the kinetics of release has not been studied [127].

#### 2.2.3. Polylysine

**Figure 6:**
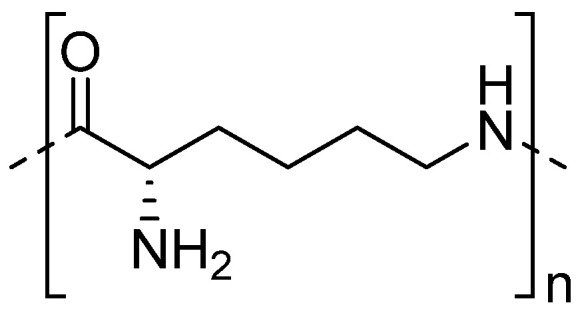
Schematic structure of polylysine.

The last protein amino acid worth mentioning is homopolypeptide—polylysine (PL) (Figure 6). PL is utilized in biomaterials due to its high safety, water-solubility, stability, as well as antibacterial properties. It is reported that this AA may also be used to induce embryonic signaling processes during chondrogenesis in cartilage tissue engineering [128]. However, there are no many published studies on its application to cure skeletal system problems. One example is a polymer carrier from PLGA used to deliver this specific biomolecule in the targeted therapy. The surface of PLGA microspheres was first modified by introducing carboxyl groups, and this way, amino acids could be immobilized. Its release in vitro in the presence of MG63 human osteoblast-like cells positively influenced their adhesion and proliferation [129]. Another variant was the modification of poly(3-hydroxybutyrate-co-3-hydroxyvalerate)-HAp/bredigite nanofibrous composite scaffolds. The functionalization of the material was carried out by two methods, the use of the sorption process and covalent attachment. The kinetics of release has not been studied; however, the results suggest the possibility of using such connections in bone tissue regeneration [130].

### 2.3. Osteopontin (Bone Sialoprotein)

Osteopontin is a protein encoded in humans by the SPP1 gene and is also known as bone sialoprotein (BSP). It plays a role in apoptosis processes, cell activation, and chemotaxis. However, it is primarily a potential nucleator of HAp, responsible for biomineralization and bone remodeling [131]. An interesting fact is that with the assistance of osteopontin or appropriate antibodies, it is possible to treat specific human pathologies, e.g., autoimmune disease, cancer metastasis, immune organ atrophy, and also, primarily important in this review, bone remodeling [132]. Therefore, the osteoconductive tissue response of a biomaterial composed of a PLA matrix functionalized with HAp nanoparticles and bone sialoprotein was investigated. As a result of in vivo testing, a localized satisfied effect caused by protein release was observed. The presence of osteopontin improved the activation of bone-forming cells [133]. Another type of ceramics used to bind BSP is calcium silicate. An osteopontin-sequenced polypeptide SVVYGLR was grafted into mesoporous calcium silicate and 3D-printed into scaffolds that positively stimulated VEGF expression to improve angiogenesis during in vivo tests [134]. As in natural conditions, HAp crystals are deposited onto the type I Col; this protein was utilized to create bone sialoprotein-Col-guided materials. These display an increase in nucleation potency, yet the effect in both cases was minimal [135,136]. Col type I was also used to create carbonate-containing apatite/Col sponges decorated with BSP. The modification occurred via immersion technique. In vivo studies showed that one week after implantation into a tissue defect in rat tibia, the migration of numerous vascular endothelial cells inside the graft was observed [137]. Other functionalization of polymer matrix includes PCL/poly(2-hydroxyethyl methacrylate) surface treatment in regulating osseous tissue formation. Immobilizing BSP enhanced the attachment of osteoblastic cells [138]. However, in other studies, in order to determine protein-specific properties, different polymer-based porous scaffolds, with the absence of a mineral component, were modified via the sorption method. The obtained results suggest that chosen non-bioactive surfaces of biomaterials, e.g., polystyrene plates, β-tricalcium phosphate coated polystyrene discs, and poly(ethylene glycol terephthalate)/poly(butylene terephthalate) films with BSP, are not sufficient to prime bone marrow stromal cells for functional osteoblastic differentiation in vivo [139]. BSP, on the other hand, works effectively as a modifier of metallic surfaces. Titanium alloys are one of the most frequently used metallic biomaterials in bone problems. A Ti femoral implant was coated with protein by the physisorption method. As a result of in vivo as well as in vitro investigations, the observed increase in calcium deposition and the stimulation of cell differentiation induced by bone sialoprotein highlight its potential as a surface modifier that could enhance the osseointegration of orthopedic implants [140].

### 2.4. Osteocalcin

**Figure 7:**
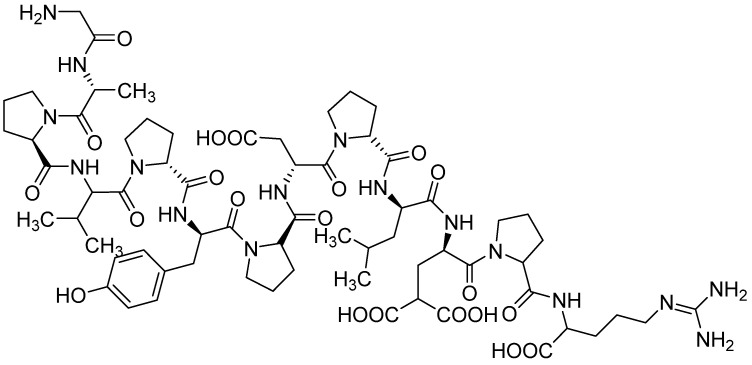
Chemical structure of osteocalcin.

In recent years, evidence has been gathered that bone functions not only as a static structural organ that supports movement but also as an endocrine organ. One of the factors released by the skeleton is osteocalcin (Figure 7) [141].

It is an osteoblast-specific secreted protein that acts as a hormone by stimulating insulin production but also demonstrates an impact on bone mineralization and density [142]. The protein has been combined with ceramic/polymer materials featuring different structures, either pastes or cylindrical implants. In the first case, the bone cement was mixed with mineralized Col, and then osteocalcin was added to the cement paste during the setting [143]. In another one, calcium phosphate cement, containing various types of ceramic, was used as a starting material as well as a base for the whole implant. After combining with Col, a fiber-reinforced material was obtained and loaded with protein [144]. Although the release kinetics have not been studied in either case, both results suggest that osteocalcin activates osteoblasts as well as osteoclasts during early bone formation.

### 2.5. Osteonectin

Osteonectin is a non-collagenous protein of bone matrix that works as a calcium-binding matricellular factor in skeletal tissue [145]. The bone skeleton function refers to the differentiation of bone cells, remodeling control, and maintenance of bone mass [146]. An effect of osteonectin-derived glutamic acid sequence on the viscoelastic properties of poly(lactide ethylene oxide-fumarate) (PLEOF)/HAp composite was investigated. This way, a model of degradable material in bone regeneration was created [147]. Another connection with HAp involves the utilization of mineralized type I Col nanofibers. During synthesis, the protein was added to the polymer solution. As a result of in vivo studies, the formation of new mineralized fibers was observed in the presence of osteonectin. This could provide new insights into the novel mineralization function of this protein for bone development in in vivo conditions [148].

**Table 1:** Summary of the discussed protein biomolecule references.

Protein Biomolecule	References
Growth factors—TGF-β	[38,39,40,41,42,44,45,46,47,48,49,50,51,52,53,54,55,56,57,58,59,60,61,62,63,64,65,66,67,68,69,70,71]
Growth factors—IGF	[44,45,46,47]
Growth factors—SDF	[48]
Growth factors—BMP	[52,53,54,55,56,57,58,59,60,61,62,63,64,65,66,67,69,71,73,74,75,76,77,78,79,80,99]
Growth factors—FGF	[67,69,101,102,103,104,105]
Growth factors—VEGF	[42,74,74,75,76,77,78,79,80,81,82,83,84,85,86,87,88,89,90,94]
Growth factors—PGDF	[94,95,96,97,98,99]
Growth factors—NGF	[107]
Protein AA—RGD	[113,114,115,116,117,118,119,120,121,122]
Protein AA—Arg	[124,125,126,127]
Protein AA—PL	[129,130]
Osteopontin (bone sialoprotien)	[133,134,135,136,137,138,139,140]
Osteocalcin	[143,144]
Osteonectin	[147,148]

## 3. Non-Protein Biomolecules

### 3.1. Hormones

Hormones play a significant role in the proper functioning of the body. They are natural chemical messengers synthesized from a specific cell group. They constitute an excellent communication system from one tissue/cell to others in the body, participate in dynamic control of biochemical and physiological functions, and coordinate many processes in biological systems (neuroendocrine and immunological control). Hormonal imbalances cause disorders of regulatory mechanisms and thus disturb the homodynamic balance [149]. Therefore, the presence of hormones in implants increases the material’s bioactivity, while the local delivery can provide a better as well as faster therapeutic effect. Based on the available literature, systems of local hormone delivery are presented.

#### 3.1.1. Parathyroid Hormone

Parathyroid hormone (PTH) is produced by the glands adjacent to the thyroid gland, which control the calcium level in blood. When calcium concentration drops, the secretion of PTH increases, as it is responsible for stimulating the formation and resorption of bone tissue. The sporadic injection of small amounts determined bone formation causing bones to become stronger. Human trials have proved these abilities, and PTH itself is already used in the therapy of osteoporosis [150,151]. Biodegradable PLGA nanoparticles containing human parathyroid hormone, prepared by a modified double emulsion-solvent diffusion-evaporation method, were loaded into porous freeze-dried CS-Gel scaffolds. In vitro tests confirmed that the released PTH was biologically active during incubation and did not lose its properties due to binding to PLGA. The controlled release was observed for 28 days [152]. PLGA was also used to produce microspheres by double emulsion technique. PTH has been encapsulated and then analyzed in vitro and in vivo in relation to laboratory mice to determine the kinetics of its release and to induce a biological reaction in the bone [153]. As polymeric matrices can easily be modified with active substances, a hydrogel matrix with PEG and RGD peptides containing covalently bound peptides of PTH was designed. The study aimed to investigate the synergic effect of the released hormone and RGD on bone tissue regeneration processes [154]. A highly porous 3D nanofibrous scaffolds made of PLLA have been used to determine the optimal kinetics of release of PTH depending on the method of administration in a local bone regeneration model. The pulse method, in which a sandwich-like composite consisting of alternate alginate-PTH layers and polyanhydride (PA) insulating layers in a PCL sealant matrix, was compared with the continuous release method. In the second model, also in the PCL sealant matrix, PA microspheres loaded with PTH were charged. The difference between these two types of devices is in PTH distribution, where PTH is distributed in a layered structure to achieve pulsatile release or more uniformly in the matrix within microspheres to achieve continuous release. The kinetics was tested in vivo on a mice model [155]. Another way of pulsed PTH administration was applying a cylindrical device fabricated with a biodegradable PLLA, using a reverse solid free-form fabrication technique. On the supplied equipment, alternating isolation layers of sebacic acid, 1,3-bis(*p*-carboxyphenoxy)propane, PEG, and layers of PTH-loaded alginate were placed. The lag time was modulated by layer composition and film thickness [156]. In order to enhance the biomimetics of the carriers, biomaterials with ceramics demonstrating bioactivity are designed. A hybrid scaffold was manufactured by immobilizing polyphosphate-functionalized nHAp (PP-nHAp) on the porous surface, followed by PTH loading on the polyphosphates of nHAp surfaces. The hormone was sustainably released for up to 50 days. The results suggest a synergistic effect of using PTH and nHAp to enhance bone healing in the animal model [157]. Bioactive ceramic is not only HAp; therefore, β-tricalcium phosphate was also used to create such biomaterial. A ceramic-polymer composite of β-TCP and Col (β-TCP/Col) was created and then combined with PTH. Obtained results demonstrate that a combination of single-dose local administration of PTH and β-TCP/Col had an additive effect on local bone formation in osteoporosis rats [158]. As hydrogels display structural similarity to natural tissues, PTH was loaded in a thiol-ene hydrogel at several concentrations and polymerized in and around an osteoconductive poly(propylene fumarate) (PPF) scaffold. The obtained biomaterial allowed the release of 80% of the hormone within 4 days, which showed bioactivity for 3 weeks [159]. PTH is only adequate when dosed by injection, because it has no oral bioavailability; therefore, a particularly interesting project was that describing the oral absorbtion of PTH in rats and monkeys facilitated by a delivery agent, 8-[(2-hydroxy-4-methoxybenzoyl)amino]caprylic acid (4-MOAC). In this study, dosing solutions were prepared by adding PTH to an aqueous solution of 4-MOAC in water. The obtained data suggest that 4-MOAC facilitates the gastrointestinal absorption of biologically active PTH in oral dosing of a 4 MOAC/PTH aqueous solution [160].

There are known combinations of biomaterials based on parathyroid hormone-related protein (PTHrP). It is used in bone-related therapy as it has been shown to induce bone anabolic actions in rodents and humans upon daily systemic administration. For this reason, an implant was created based on Gel-glutaraldehyde biopolymer-coated nanocrystalline HAp. These macroporous foams were then modified with PTHrP and biologically tested [161]. Osteostatin (OSN) is a fragment of PTHrP. ONS was chemically immobilized on a Col–HAp scaffold. The chemical attachment method via crosslinking ensures controlled continuous release in time. The in vitro and in vivo results confirmed that such a system may be adopted for a range of different proteins and thus offers the potential for treating various complex pathologies that require localized mediation drug delivery [162]. Apart from pure PTH, biomedicine also uses a PTH-derived peptide, PTHdP. Its potential as a bone growth factor for bone tissue engineering and bioactivity in the biological environment after being incorporated into the nHAp/CS scaffold was evaluated. It was demonstrated that PTHdP could significantly promote or inhibit osteogenesis when exposed intermittently or continuously to MC3T3-E1 cell culture. Moreover, sustained and controlled release of the bioactive molecule was achieved [163].

#### 3.1.2. Vitamin D3

**Figure 8:**
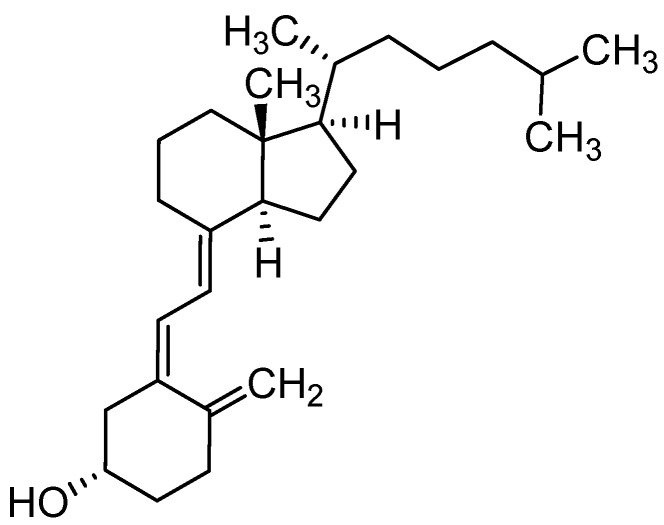
Structure of cholecalciferol.

It is well known that vitamin D3 (cholecalciferol, Figure 8) plays a crucial role in remodeling and maintaining proper bone condition [164]. The active form of vitamin D3 (VitD3) used in biomedicine is calcitriol (Cal), produced by its hydroxylation in the liver by 25-hydroxycalciferol hydroxylase [165].

The biomolecular-enabled coating was designed to improve osteogenic capabilities in bone tissue engineering. Polyelectrolyte multilayered (PEM) film coating with local immobilization of Cal in BCP scaffolds to promote osteoporotic bone regeneration by targeting the calcium-sensing receptor was designed [166]. Another ceramic/polymer material is a nanocomposite scaffold manufactured by electrospinning. The polymer scaffold, forming matrices, was made of PCL/Gel, reinforced by nHAp, and subsequently modified with VitD3. MG-63 cell line was cultured on the manufactured composite scaffolds, and the results confirmed a positive influence on the proliferation of cells with this form of Cal [167]. Subsequent studies of the same scaffold confirmed the possibility of a smooth release of VitD3in time [168]. As the main limitation of Cal is the short half-life in the bloodstream, new therapeutic strategies and solutions are being sought to overcome this barrier. Using the single emulsion solvent evaporation method, VitD3 was encapsulated in PLGA nanoparticles. As a result, the nanoparticles remained stable under storage conditions for several weeks, and they were successfully lyophilized to increase their shelf-life using a cryoprotectant [169]. Porous scaffolds made from PLGA loaded with Cal were designed using the same components. These fully absorbable osteogenic biomaterials were prepared using the solvent casting/salt leaching method [170]. Specific scaffolds were used to investigate the influence of Cal on osteoinduction following local administration into mandibular bone defects. Hormone-loaded absorbable Col membrane scaffolds were prepared by the polydopamine coating method. Following in vivo implantation, Cal-loaded composite scaffolds underwent rapid degradation compared to materials without hormones, with pronounced replacement by new bone layers [171]. Absorbable Col fleece was soaked in Cal solution and then implanted in bone defects in the maxilla and mandible of rats. There was no significant difference between the control and research groups; moreover, the kinetics of hormone release was not investigated [172]. In order to improve the bioavailability of VitD3, it was essential to increase its solubility in water. Therefore, oleoyl alginate ester (OAE) hydrophobic nanoparticles served as hormone carriers and were prepared by acid chloride reaction. This resulted in oral carriers penetrating through cell walls and demonstrating the permanent release of the biomolecules [173].

#### 3.1.3. Calcitonin

The hormone with an important role in the treatment of osteoporosis is calcitonin. It is produced by the thyroid gland and influences the inhibition of bone resorption, and the reduction of osteoclast formation has been proven [174]. A biomaterial was designed in which salmon calcitonin (sCT) was combined with pentapeptide-decorated silica nanoparticles (SiO_2_ PepsCT). The purpose of the biomaterial was to improve therapeutic effectiveness. In vivo studies showed that the material also affects the extension of the sCT half-life [175]. Another sophisticated project was a complex of sCT and oxidized calcium alginate (sCT-OCA) loaded into a thermosensitive copolymer hydrogel. A polymer matrix consisted of PLGA-*b*-poly(ethylene glycol)-*b*-PLGA (PLGA-PEG-PLGA). Due to the nature and properties of the biomaterial, the sustained release of sCT was determined by the degradation of the hydrogel as well as the decomposition of the sCT-OCA complex [176]. In a ceramic-polymer carrier, PLGA and cement with calcium phosphate (CPC) were used. In order to obtain such a composite, microspheres with PLGA were loaded sCT and then incorporated into CPC. The hormone’s release rate depended on the amount of the polymer phase [177]. A ceramic mixture containing 70% HAp and 30% β-TCP was modified with calcitonin by immersion in a hormone solution. As a result of in vitro and in vivo studies, an increase in the degree of osteogenesis was observed [178]. A similar method involving the modification of ceramic by sorption was used to produce nHAp loaded with sCT to obtain sCT-HAp-NPs [179]. Apart from the described hormone, there are connections with calcitonin gene-related peptides (CGRP). It was used as an osteogenic factor to modify a hydrogel scaffold composed of HAp and sodium alginate (SA), manufactured by the 3D printing method. Materials displayed active proliferation and differentiation in vitro and in vivo, highlighting osteoinductive abilities [180].

#### 3.1.4. Estrogen and Testosterone

**Figure 9:**
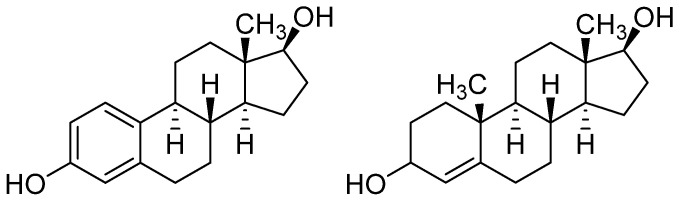
Structure of estradiol and testosterone.

Not only the hormones responsible for regulating calcium levels are essential in controlling skeletal growth and maintaining bone mass as well as strength, but also sex hormones. Both female estrogen and male testosterone (Figure 9) affect the bone in men and women [181]. The relation between changes in bone density and estrogen level is well known. Changes in hormonal economy after menopause, particularly in estrogen expression, are the leading cause of osteoporosis [182]. However, the quantity of testosterone is equally crucial for a healthy skeleton, as it has been shown to directly affect bone cells and bone metabolism [183]. The ability to improve osteogenic differentiation of human bone marrow mesenchymal stromal cells in the presence of estrogen was investigated by introducing this hormone on biodegradable PLGA microparticles. They ensured the intracellular release of biomolecules for seven days, which enabled the effective regulation of MSCs [184]. Estradiol, a primary natural estrogen, has been loaded into PCL/SF microfibers obtained by the electrospun method. The addition of SF increased the bioactivity of the polymer matrix. The sustained release of the hormone from the biomaterial lasted for about three weeks [185]. Its encapsulation provided impressive results regarding estrogen release in PLGA nanoparticles produced via an emulsion-diffusion-evaporation method. Hormone-loaded PLGA NPs were then placed in macroporous HAp-CS scaffolds. The nanoparticles were bonded to the scaffolds in two ways. The first method using embedding ensured the release of biomolecules for 55 days, whereas in scaffolds loaded during manufacture, the controlled release behavior of estradiol was observed for over 135 days [186]. The interesting idea was to use nanodiamond (ND) particles as a carrier. The estrogen-ND complex was then loaded into a photo–crosslinkable methacrylate glycol CS hydrogel (G). The prepared estradiol/ND/G platform increased the beneficial impact of estrogen through extended release with the highest efficiency and safety at a local level [187].

Porous biomaterials were obtained from mesoporous bioactive glass modified with testosterone. In order to obtain these scaffolds, the solutions of both components were thoroughly mixed and then freeze-dried. Sustained-release testosterone was confirmed by the results obtained [188]. A load-bearing biodegradable scaffold made of polypropylene fumarate/tricalcium phosphate composites was modified by adding testosterone, bone morphogenetic protein-2 (BMP-2), or their combination in order to promote bone regeneration. The obtained results demonstrated that testosterone is as effective as BMP-2 in promoting the healing of critical-size segmental bone defects and that combination therapy with testosterone and BMP-2 is superior to single therapy. However, the kinetics of the release of the compounds has not been investigated [189]. The influence of the male sex hormone on cell proliferation and differentiation has been examined in relation to ceramic-polymer scaffolds based on PCL/BCP. These materials were modified with the addition of PLGA as well as testosterone (T) resulting in two types of composites, PCL/BCP/T and PLGA/PCL/BCP/T. Both scaffolds were associated with desirable characteristics for bone tissue applications; however, alkaline phosphatase levels expressed by osteoblasts were significantly greater with PLGA/PCL/BCP/T [190].

#### 3.1.5. Insulin

**Figure 10:**
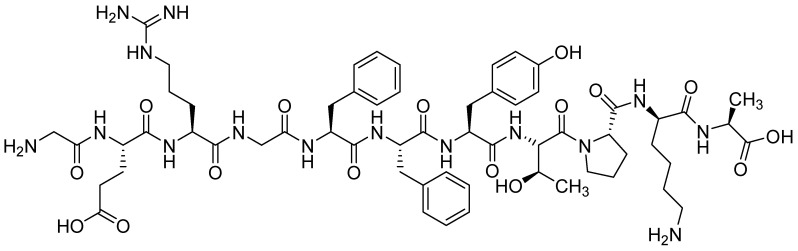
Schematic illustration of the chemical structure of insulin.

Clinical and experimental studies show that insulin (Figure 10), a peptide hormone produced by the pancreatic islands, is closely related to bone density. Using it as an anabolic agent can preserve and increase bone strength through its effects on bone formation [191]. Different types of biomaterials are designed for the local supply of this biomolecule. In a biomimetic one, insulin was chemically grafted onto the surface of HAp nanorods (nHA). The insulin-grafted nHAs (nHA-I) were dispersed in PLGA polymer solution, which was electrospun to prepare PLGA/nHA-I composite nanofiber scaffolds. The obtained results suggest that the PLGA/nHA-I composite nanofiber scaffold can enhance osteoblastic cell growth, as more cells were proliferated and differentiated. However, the carrier’s hormone release rate has not been studied [192]. Similar organic/inorganic materials enriched with Col have been manufactured. Insulin-loaded PLGA particles were incorporated into porous nHAp/Col scaffolds. In vitro and in vivo studies confirmed that the bioactive hormone was successfully released from the PLGA particles within the scaffold, and the size of the particles as well as the release kinetics of the insulin could be efficiently controlled. Furthermore, the biomaterials significantly accelerated bone healing [193,194]. Scaffolds containing systematic gradients mimicking the significant gradients observed in native tissues were designed. Nanoparticles of insulin and β-glycerophosphate (β-GP) were incorporated into a non-woven mat of PCL. Human adipose-derived stromal cells (hADSCs) were cultured on these graded non-woven mats to probe their effects on the development of cellularity and mineralization. The obtained results showed that the differentiation of the stem cells increased at insulin-rich locations [195]. Instead of pure insulin, insulin-like growth factor I (IGF-I) is also often used in research. It is entrapped in the mineralized matrix of the bone during formation and affects cell proliferation by stimulating growth in various progenitor cell types. Thus, controlled IGF-I-releasing SF scaffolds were designed [196]. Another biomimetic project involving the use of ceramics consisted of the manufacture of scaffolds from alginate, tricalcium phosphate (TCP) granules, and PLGA microspheres (MS) loaded with osteoinductive IGF-I. Controlled and sustainable IGF-I release was observed for 28 days [197]. In another study, to accelerate the deposition of bone-like minerals (BLM) on the surface of the designed biomaterial, three-dimensional (3D) PLGA porous scaffolds have been modified by applying surface treatments. PLGA was incubated in an SBF solution enriched with IGF-I. Obtained mineralized scaffolds demonstrated slow controlled release over a 30-day period when they were incubated in phosphate-buffered saline (PBS) at 37 °C. Increased proliferation of bone marrow stromal cells was observed in in vitro as well as in vivo studies [198].

### 3.2. Flavonoids

**Figure 11:**
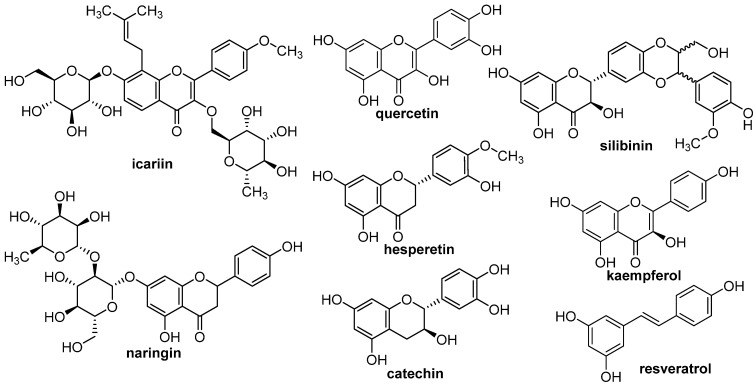
Chemical structure of discussed flavonoid-based compounds.

Flavonoids are a broad group of organic chemicals found in plants. They serve as dyes, antioxidants, and natural insecticides as well as fungicides, protecting against insect and fungal attacks [199]. However, due to their specific nature, they also demonstrate interesting biochemical and antioxidant effects associated with many diseases, such as cancer, arteriosclerosis, and Alzheimer’s disease [200]. They are components of many pharmaceuticals and cosmetics, considering their interesting anti-carcinogenic, antioxidative, anti-inflammatory, and anti-mutagenic properties [201]. In the regeneration of the skeletal system, these non-protein biomolecules are particularly important in stimulating the osteoblastogenesis process, which leads to the formation of new bone layers by supporting the differentiation of MSCs into osteoblasts [202]. Based on the available literature, this part of the review will briefly discuss approaches to the delivery of selected flavonoids, such as icariin, resveratrol, quercetin, and others (structures presented in Figure 11) that have been proven to be proregenerative in bone tissue.

#### 3.2.1. Icariin

In the regeneration of bone tissue with proven osteogenic and angiogenic effects, icariin (ICA) is an essential flavonoid. Several reports have shown that ICA (Figure 11) can inhibit osteoclast differentiation and increase osteogenic differentiation of mesenchymal bone stem cells (BMSCs) [203]. In order to provide the implants with the greatest possible biomimetics, they are bound to ceramics, which makes the resulting structure relatively similar to natural bone tissue. Bioactive scaffolds strengthening bone repair were developed by loading ICA into porous scaffolds with tricalcium phosphate (TCP), and the obtained porous Ica/TCP composites were then investigated for treating osteonecrosis of the femoral head (ONFH) in a rabbit model [204]. A bioactive PLGA/calcium phosphate/ICA (PTI) scaffold was fabricated by an innovative low-temperature 3D printing technology. Potentially, the main use of this biomaterial is to treat steroid-associated osteonecrosis. The obtained and presented results based on in vitro and in vivo tests against rabbit models suggest that the scaffolds are a promising potential strategy for bone tissue engineering and regeneration in patients with challenging bone cancer [205]. HAp ceramics was also used to create composites with this flavonoid. Micro/nHAp granules were modified with ICA by sorption process, consisting of immersing them in a flavonoid solution and freeze-drying [206]. Similar systems containing additional CS, a natural, degradable polymer that increases the ability of bone precursor cells to differentiate and promotes the formation of new bone tissue, are also known. The scaffolds were generated by thoroughly mixing ICA and CS/HAp in micro- as well as nanoscale (ICA-CS/HA) using the freeze-drying technique. In all cases, the drug release behavior has demonstrated that the ICA loading CS/HA scaffolds can achieve the basic effect of the permanent release of the drug simultaneously with a satisfactory effect on the bone tissue regeneration processes [207,208,209]. More advanced systems have also been described. Composite biomaterials were designed via the electrospun method, using Col, PCL, and HAp, additionally containing CS microspheres in which the ICA was encapsulated [210]. Combining a natural polymer, water-soluble carboxymethyl CS (CMCS) with a synthetic oil-soluble PLGA, nHAp-reinforced hybrid scaffolds loaded with ICA (ICA-loaded nHAP/CMCS/PLGA) were developed. As a result, an implant with a topological structure similar to natural bone was created. Based on in vivo and in vitro results, scaffolds effectively promoted the osteoblasts’ adhesion, proliferation, and differentiation, thus having great potential and providing a unique strategy for bone repair and regeneration [211]. Another biopolymer known to be used in drug and cell delivery systems is alginate. In its spheres, ICA was enclosed with and then combined with HAp. The freeze-drying method was used to obtain a HAp/alginate (HAA) porous composite scaffold loaded with flavonoids. The obtained data suggest its promising application in biomedicine as it mediates the processes of coupling of osteogenesis induction and inhibits osteoclast activity [212]. Bioglass-based materials are also manufactured. Using the foam replication technique, the Gel-coated 3D sponge-like scaffolds based on 45S5 bioactive glass were created. The prepared implants exhibited slightly different properties and the kinetics of flavonoid release, depending on the crosslinking agent applied, caffeic acid or EDC/NHS ((3-dimethylaminopropyl)-*N*′-ethylcarbodiimide hydrochloride/*N*-hydroxysuccinimide) [213]. Hybrid porous ICA-loaded hollow bioglass/CS (ICA/HBG/CS) therapeutic scaffolds were developed to treat critical-sized bone defects [214]. Carriers without a ceramic phase mainly focus on using the characteristic polymer structure to deliver the active substance. Mg^2+^ ions were used as a carrier. Hydrophobic ICA was preloaded on MgO/MgCO_3_ molecules and then enclosed in microspheres made out of PLGA, which is biodegradable in the biological environment. Such a degradable system allows double-controlled release [215]. PLLA was used to create fibrous membranes using the electrospinning method. An intermediate layer of PDA was then applied to them in order to obtain increased cytocompatibility as well as osteogenic activity. The membrane was functionalized with ICA to obtain PLLA-PDA-ICA biomaterial [216]. The fibrous membrane, which simulates the artificial periosteum, was made by the electrospun method. For this purpose, ICA was introduced into PCL/Gel nanofibers [217]. Increased bone cell proliferation was achieved by using porous scaffolds. Biomaterials were made by combining the solvent casting and salt leaching techniques from poly(3-hydroxybutyrate-co-3-hydroxyvalerate) (PHBV). This way, novel ICA delivery porous PHBV scaffolds (IDPPSs) were fabricated [218]. HA is used in tissue engineering and biomaterials due to its high hydrophilicity as well as a positive effect on chondrogenesis of stem cells and chondrocytes, cartilage formation, and the integration of the neocartilage with the surrounding native cartilage. These properties are significant for the regeneration of bone and cartilage tissue. An HA-ICA hydrogel was designed. Methacrylic anhydride (MA) was combined with flavonoid and then dissolved in a solution of MA-modified HA, which resulted in obtaining the hydrogel [219]. The three-component system, enriched with Gel, was made by an emulsion-coagulation method using glutaraldehyde (GA) as a crosslinking substance. Composite microspheres of Gel/hyaluronic acid (Gel/HA) loaded with ICA as a controlled release system were presented. The study showed that the rate of release of ICA from the microspheres can be relatively easily modified by changing the GA content and crosslinking time [220]. Similarly, hydrogel materials were obtained from ICA conjugated with HA/Col (Ica-HA/Col) to promote osteochondral interface restoration [221]. Due to the impressive properties of nanomaterials and nanoparticles, there is a growing interest in their use in biomedicine. TiO_2_ nanotubes were used in the study, as they are an interesting component in biomaterials for bone tissue regeneration. It was demonstrated that the surface morphology of TiO_2_ nanotubes could improve the adhesion, proliferation, and differentiation of osteoblast cells and MSCs. Therefore, an ICA-functionalized coating composed of ICA and PLGA on the TiO_2_ nanotube surface (NT-ICA-PLGA) to promote osteoblast cell activity and early osseointegration was designed [222]. A composite structure consisting of ICA-loaded and CS-Gel-sealed TiO_2_ nanotubes was created to control the drug release profile and improve the biocompatibility of Ti substrates. Firstly, the flavonoid was placed in the TiO_2_ nanotube space and then sealed with CS-Gel multilayer coatings. Based on obtained results, it is suggested that such a nanotube structure can modulate the bioactivity of primary osteoblasts [223]. A small intestine submucosa (SIS) was used as an excellent, natural biological carrier. However, SIS provides a different microenvironment than bone tissue; thus, its structure was modified with ICA. This has resulted in a permanent SIS scaffold with improved osteoinductivity and controlled local delivery of ICA. Implants were in vivo tested in a mouse calvarial defect model, and the results of this study suggest that the SIS scaffold has the potential as an ICA delivery carrier for the enhancement of bone regeneration [224].

#### 3.2.2. Quercetin

A particularly important place in the regeneration of the skeletal system is occupied by quercetin (QU, Figure 11). It was demonstrated that compared with other flavonoids, for example, kaempferol, it has a better stimulating effect on osteogenic differentiation of hADSC and is helpful for in vivo bone engineering [225].

As QU is poorly absorbed during oral administration, QU-loaded phytosome nanoparticles (QPs) have been prepared using the thin film hydration method. QP exhibited very high encapsulation efficiency (98.4%), and the results confirmed its superiority over free QU at the same doses as a promising hormone replacement therapy [226]. The scaffold, demonstrating a very beneficial effect on the proliferation and attachment of MC3T3-E1 cells and promoting the expression of related genes and osteogenic proteins, was made with 3D-printed PLLA. The polymer was functionalized by QU with a layer of PDA and then analyzed biologically in relation to MC3T3-E1, an osteoblast precursor cell line derived from mouse calvaria. As a result, the potential of the 3D-printed QU/PD-PLLA scaffolds with a certain amount of flavonoid as a bone-repair material was confirmed [227]. With good effects, it was decided to modify the obtained QU/PD-PLLA scaffold with CS to give it specific properties. In this way, PLLA/CS-D/QU was obtained. First, the 3D-printed PLLA scaffold was incubated in a CS solution and then freeze-dried to obtain PLLA/CS scaffolds with micro/nano-fiber hierarchical structure [228]. In order to imitate the natural bone in the best possible way, QU was also combined with ceramics. A simple two-component nHAp/QU composite was proposed. Vacuum freeze-drying technology was used to fabricate the drug delivery system. The flavonoid was mixed with the nHA bioceramic microspheres, and modification occurred through the sorption process [229]. A more advanced three-component structure built of an SF/HAp scaffold inlaid with QU (QU/SF/HAp) at different concentrations promoted osteogenesis, mainly focusing on QU’s ability to enhance bone health. QU was loaded on the SF/HAp scaffolds using freeze drying [230]. Composites containing various biomaterials of natural origin were also proposed. Col obtained from duck feet was used as a polymer phase of the scaffold composed of QU and HAp (QU/DC/Hap), improving osteoconductive properties. Obtained QU/DC/HAp sponges were tested in vitro in relation to BMSCs as well as in vivo against rat calvarial bone defects. The results confirmed that these bioengineered sponges could improve bone tissue regeneration [231]. Another material of natural origin used for the biometric scaffold was goat lung fabricated by decellularization of lung tissue. After appropriate treatment, it was modified by crosslinking with QU and nHAp and characterized to evaluate the suitability of the QU crosslinked nHAp-modified scaffold for the regeneration of bone tissue [232].

#### 3.2.3. Naringin

Naringin (Figure 11) is a flavonoid demonstrating therapeutic effects in diseases related to bone metabolism [233].

Modifying a biodegradable composite with this flavonoid determined its potential to repair bone defects. A porous composite containing genipin crosslinked Gel and *β*-tricalcium phosphate (GGT) was fabricated by the salt-leaching method to carry naringin (GGTN). The ability to regenerate bone tissue in vivo was assessed using the biological response of the rabbit calvary bone to these materials. Higher growth of new apatite layers at the site of GGTN implantation than of simultaneous GGT implantation was demonstrated; therefore, GGTN is promising as a bone substitute [234]. Naringin has also been supplied by incorporation into electrospun nanoscaffold containing PCL and PEG-*b*-PCL. This allowed for the creation of PCL/PEG-*b*-PCL/naringin nanoscaffolds. Based on the critical size defect (CSD) model of mouse calvarial bone and the presented results, it was shown that the scaffolds strengthen osteoblast functions and suppress osteoclast formation [235]. The electrospinning method was also used to create naringin-loaded microsphere/sucrose acetate isobutyrate (Ng-m-SAIB) hybrid depots to improve osteogenesis in the calvarial defects of SD rats. Demonstrated results confirmed that Ng-m-SAIB hybrid depots might have promise in bone regeneration applications [236].

#### 3.2.4. Silymarin

Silymarin (a standardized extract from the seeds of *Silybum marianum*) is composed of many compounds, with silibinin (Figure 11) being its main active ingredient. Silymarin (SIL) supports osteoblast proliferation, inhibits osteoclast proliferation, and positively affects bone regeneration. It was combined with HAp with good osteoclastic properties. For this purpose, titanium plates were covered with ceramics and then incubated in modified SBF with SIL to be absorbed. This way, titanium implants with SIL-loaded HA coatings were obtained and then implanted in twelve-week-old female Sprague Dawley rats. The results suggest that the local incorporation of coatings with SIL is helpful in improving new bone formation around the surface of titanium rods [237]. Another combination of SIL with HAp presupposed the formation of Col sponges from the Col of natural origin from duck feet (DC) by freeze-drying. The in vitro results against rabbit bone marrow stem cells and in vivo results in a rat calvarial defect model confirmed that Smn/DC/HAp grafts support cell adhesion, proliferation, and osteogenicity [238].

#### 3.2.5. Hesperetin

Hesperetin (Figure 11) was supplied by a Gel sponge in the rat model hMSC using a scaffold combined with hesperetin/Gel. This model has influenced the rate of healing of tibial fractures in rats, speeding up the process [239].

#### 3.2.6. Kaempferol

The kaempferol (Figure 11) was delivered in a layered composite LBL (layer by layer)-kaempferol composed of alternating layers of sodium alginate and protamine sulfate on the CaCO_3_ core. The drug release rate was determined by its concentration in the biomaterial and layer thickness. However, it was shown that the demonstrated method enhanced drug delivery and improved pharmacokinetics [240].

#### 3.2.7. Catechin

The influence of catechin (CC, Figure 11) on osteogenesis and mineralization has been investigated based on coatings modified with catechin hydrate. As this flavonoid is a simple multifunctional material-independent coating compound, its modification was performed by the dip-coating method. The selected media were polystyrene, silicon oxide, titanium oxide, PCL nanofiber, glass coverslip, gold, polytetrafluoroethylene, and polydimethylsiloxane. In vitro tests were performed on human adipose-derived stem cells (hADSCs) and human umbilical vein endothelial cells (HUVECs), and in vivo on a mouse calvarial defect model. As a result, it was concluded that CC-based media not only improved cell adhesion and proliferation but also significantly improved osteogenesis of hADSCs in vitro and in vivo due to the intrinsic biochemical properties of CC, including antioxidant and high calcium binding affinity [241]. Taking into account the impressive properties of CC and the effect of selenium on bone strength, a nanocomposite was formed by modifying the nHAp with this element and then combining them with CC/SE-HAp. The addition of the flavonoid was intended to improve the anticancer activity of Se-HAp nanoparticles against osteosarcoma. Studies were carried out on two lines of human cells, normal bone marrow stem cells (hBMSCs) and human osteosarcoma cell lines (MNNG/HOS). The study showed that combining a natural biomaterial (i.e., CC) with Se and HAp may be a practical therapeutic approach in bone cancer therapy [242]. The combination of CC and mesoporous HAp (mHAp) was possible through a stable amide connection resulting from the previous modification with (3-aminopropyl)triethoxysilane. Then the short- and long-term responses of cultured MSCs, osteosarcoma cells (Saos-2), and doxorubicin-resistant cells (RSaos-2/Dox) on the surface of the prepared Cat@MHAp biomaterials were investigated. Based on the results, it was found that Cat@MHAP decreases the proliferation of Saos2 and RSaos-2/Dox cells in a time-dependent manner. At the same time, it supports the growth of MSCs, indicating the ability of Cat@MHAP to distinguish tumor cells from normal ones [243]. Epigallocatechin gallate (EGCG) is a type of the most abundant green tea CC [244]. The effect of the combination of EGCG and α-TCP on the bone regeneration capacity in a bilateral rat calvarial bone defect model was investigated. Modifying ceramics with the flavonoid was performed by sorption, possibly due to the porous nature of α-TCP. This study demonstrated the bone-promoting effect of the local application of EGCG using the obtained biomaterial [245]. The potential of three-component epigallocatechin gallate/duck feet Col/HAp (EGCG/DC/HAp) composite sponges obtained by freeze-drying for bone repair has been investigated. In vitro results against bone marrow-derived mesenchymal stromal cells as well as in vivo results in nude mice confirmed that EGCG/DC/Hap is able to direct osteogenic differentiation; thus, it could be applied to the human body substitute as a natural material for bone regeneration [246].

#### 3.2.8. Resveratrol

Resveratrol (RSV) is an antioxidant and anti-inflammatory polyphenol, whose beneficial therapeutic effects in type II diabetes, cardiovascular diseases, and hypertension have been proved [247].

However, more and more studies are focusing on its potential use in skeletal as well as cartilage therapy. A biodegradable RSV-loading synthetic polymer (PLA) and biopolymer (Gel) composite 3D nanoscaffold was designed to support the treatment of cartilage defects. During in vivo and in vitro research, primary chondrocytes and cartilaginous tissue cells were successfully cultured on the scaffold [248]. A bioactive RSV-PLA-Gel porous nanoscaffold was designed using the same compounds and electrospinning, freeze-drying, and uniform dispersion techniques to repair articular cartilage defects. As a result, it was found that the biomaterial promotes the repair of cartilage injury as a whole and might function via activation of the PI3K/AKT intracellular signal path [249]. The osteogenic effect of an RSV-conjugated PCL scaffold was evaluated in mesenchymal cell culture and a rat calvarial defect model. A flavonoid was coupled through a hydrolysable covalent bond with the carboxylic acid groups in a porous PCL surface grafted with acrylic acid. It was found that the incorporation of RSV caused increased alkaline phosphatase activity of rat bone marrow stromal cells and enhanced mineralization of the cell–scaffold composites in vitro [250]. In order to optimize the therapeutic effect of the flavonoid, it was inoculated into polyacrylic acid to obtain a macromolecular drug, PAA-RSV, which was then incorporated into atelocollagen hydrogels (Coll) to produce Coll/Res scaffolds. Coll/PAA-RSV scaffold was implanted into the osteochondral defect of rabbits, and, after a certain time, exhibited anti-inflammatory activity. The biomaterial was also able to remove free radicals and thus protect chondrocytes and BMSC from damage; thus, it represents a potentially significant advancement in clinical options for osteochondral damage repair [251]. Col scaffolds and human adipose tissue stem cells were combined with regenerating oral mucosa and calvarial bone with RSV. The effect of these Col/RSV scaffolds in vitro and in vivo on healing and bone regeneration was evaluated. Obtained results suggest that Col/RSV scaffolds can provide helpful biological cues that stimulate craniofacial tissue formation [252]. In order to compose the most biomimetic material possible, hydrogel reinforced with a ceramic phase was used. Based on the crosslinking reaction, a hydrogel with 3,6-anhydro-α-l-galacto-β-d-galactane modified with nHAp and RSV was obtained. Based on the physiochemical and biological analysis, it was demonstrated that the designed hydrogel is a material of biological relevance and great pharmacological potential as a carrier for bioactive compound delivery [253].

### 3.3. Lipids

It is well known that some lipids are able to influence the molding of HAp under in vivo conditions [254]. They work as in vitro and in vivo promoters of ceramic layer formation [255]. Moreover, when administered locally, they may also cause a therapeutic effect on surrounding tissues. Therefore, they are used as a part of implants as well as biomaterials that work as carriers of biomolecules. Phosphatidylserine (PS) is a quantitatively minor membrane phospholipid that plays a crucial role in cell cycle signaling, specifically in relation to apoptosis [256]. The potential of bone repair in rat calvarial defects using a combination of HAp with phosphatidylserine liposomes was investigated. The positive effect of PS on osteogenesis processes was confirmed by in vitro and in vivo studies. Mineralization occurred faster in the presence of a two-component (HAp-PS) system [257]. To imitate biological conditions similar to natural bone, phosphatidylserine was combined with Col solution, and then nHAp was added. The freeze-drying method was used to produce a porous, organic-inorganic (nHAp-Col-PS) scaffold [258]. Based on in vitro results, the release kinetics of PS correlated with the material structure [259]. The ceramic phase was also used as bioactive glass in combination with Col microspheres loaded with phosphatidylserine to receive porous scaffolds. Obtained in vivo results demonstrate the usefulness of PS for inducing enhanced bone formation in relation to Sprague Dawley rats [260] and rabbits [261]. In another similar project, such a three-component system was additionally enriched with steroidal saponins, which were also loaded in Col microparticles. A gradient, porous scaffold was obtained, and the release rate of the biomolecule was gradient-dependent [262]. Another implantological approach was to use titanium rods. Porous Ti foam was obtained by the plasma-sprayed method, and HAp coatings on its surface were also applied by plasma spraying. The layout was modified with PS by dip-coating into phospholipid solutions in chloroform [263]. Titanium was also used to create coated discs. On Ti discs, an organic matrix containing Col and phospholipid was deposited by the Langmuir-Blodgett technique. The selected phospholipid was 1,2-dipalmitoyl-sn-glycero-3-phosphatidylcholine (DPPC), because phosphatidylcholine groups are abundant in the natural cell membrane. The obtained results suggest that Col incorporation into DPPC induced the formation of biomimetic HAp nanoparticles that resembled the HAp nanoparticles found in natural bone [264]. DPPC was also used to modify mica, a mineral classified as a silicate. The film layers were composed using the commercial Langmuir–Blodgett method. Phospholipid was released in an artificial biological environment, and after a certain time, it was found that calcium phosphate minerals may precipitate on the mica surface placed in SBF [265]. A biomaterial composed of the same components was also enriched with phospholipase A2 [266]. A valuable multi-modal platform in bone tissue therapy was designed by a combination of nHap and lipid membrane mimetic coatings (LMm); the platform consisted of 69.3% phosphatidylcholine, 9.8% phosphatidylethanolamine, 2.1% lysophosphatidylcholine, and 18.8% fatty acid. Multilamellar vesicles (MUVs) were made to provide a higher therapeutic effect using the thin-film hydration method. Lipid vesicles were the carrier for the local transport of ibuprofen and ciprofloxacin. [267].

However, not only one lipid can be used to compose such biomaterials. HAp/Col scaffolds were modified with liposomes, which were prepared from a mixture of cholesterol, 1,2-distearoyl-sn-glycero-3-phosphatidylcholine (DSPC), 1,2-distearoyl-sn-glycero-3-phosphoethanolamine-n-[methoxy(polyethylene glycol) (DSPE-PEG), and a bone-binding bisphosphonate (BP) attached. The scaffold was modified with biomolecules by incubating them in a solution containing liposomes. The release rate of PEG-liposomes and BP-liposomes from the scaffolds was investigated for 7 days. Furthermore, the interior of created liposomes worked well during in vivo examination as a drug carrier [268].

**Table 2:** Summary of the discussed non-protein biomolecule references.

Non-Protein Biomolecule	References
Hormones—PTH	[152,153,154,155,156,157,158,159,160]
Hormones—PTHrP	[161,162]
Hormones—PTHdP	[163]
Hormones—vitamin D	[166,167,168,169,170,171,172,173]
Hormones—calcitonin	[175,176,177,178,179,180]
Hormones—sex hormones	[184,185,186,187,188,189,190]
Hormones—insulin	[192,193,194,195]
Flavonoids—icariin	[204,205,206,207,208,209,210,211,212,213,214,215,216,217,218,219,220,221,222,223,224]
Flavonoids—quercetin	[226,227,228,229,230,231,232]
Flavonoids—naringin	[234,235,236]
Flavonoids—silymarine	[237,238]
Flavonoids—hesperetin	[239]
Flavonoids—kaempferol	[240]
Flavonoids—catechin	[241,242,243,244,245,246]
Flavonoids—resveratrol	[248,249,250,251,252,253]
Lipids	[257,258,259,260,261,262,263,264,265,266,267]
Liposomes	[268]

## 4. Conclusions and Perspectives

This review constitutes a collection of papers representing approaches in which tissue engineering seeks to solve problems involving bone tissue. A wide range of protein and non-protein biomolecules as active components of ceramic and polymer biomaterials are discussed in detail. According to the presented benefits of the biomolecules summarized in this review, it can be concluded that they have the potential to be adapted to the patient’s specific condition. Both the type, the dose, and the delivery method of the selected molecules will affect bone regeneration and, most importantly, the patient’s condition. It is important to emphasize that biocompatible polymeric materials or polymer-based composites are most widely used as active substance carriers. These materials are generally relatively cost-effective and easy to modify, and considering their physicochemical nature, they can be easily adapted to perform specific functions (i.e., by selecting hardness, degree of cross-linking, or mass). There is no single substance, protein, or drug designed strictly for bone tissue regeneration. For this reason, the review describes many biomolecules of both protein and non-protein origin. Although osteopontin, osteocalcin, or BMP-2 by their actions definitely contribute to bone regeneration, by far the better antioxidant properties are exhibited by flavonoids. Therefore, depending on the specific action of the carrier and therapeutic effect, carriers modified with the most equal biomolecules are studied, as they exhibit various effects and impacts on the body.

This article focuses primarily on the use of drug-delivery systems for bone tissue regeneration. However, biomolecule delivery systems are also used in the treatment of heart diseases and cancer as well as in the creation of new tissues. As science advances in the field of biomaterials, it is possible to design increasingly advanced next-generation materials. The systems described in this review have great potential to be used as carriers of active substances. Nevertheless, it should be emphasized that a collaboration of chemistry, biology, physics, and medical experts is needed to perfect them. In this way, the ultimate goal of improving the health and well-being of society will be achieved.

## Data Availability

Not applicable.
